# Reverse Signaling of Tumor Necrosis Factor Superfamily Proteins in Macrophages and Microglia: Superfamily Portrait in the Neuroimmune Interface

**DOI:** 10.3389/fimmu.2019.00262

**Published:** 2019-02-19

**Authors:** Won-Ha Lee, Donggun Seo, Su-Geun Lim, Kyoungho Suk

**Affiliations:** ^1^BK21 Plus KNU Creative BioResearch Group, School of Life Sciences, Kyungpook National University, Daegu, South Korea; ^2^BK21 Plus KNU Biomedical Convergence Program, Department of Pharmacology, School of Medicine, Brain Science & Engineering Institute, Kyungpook National University, Daegu, South Korea

**Keywords:** tumor necrosis factor superfamily, immunity, inflammation, macrophage, microglia, neuroinflammation, neuroimmune interface

## Abstract

The tumor necrosis factor (TNF) superfamily (TNFSF) is a protein superfamily of type II transmembrane proteins commonly containing the TNF homology domain. The superfamily contains more than 20 protein members, which can be released from the cell membrane by proteolytic cleavage. Members of the TNFSF function as cytokines and regulate diverse biological processes, including immune responses, proliferation, differentiation, apoptosis, and embryogenesis, by binding to TNFSF receptors. Many TNFSF proteins are also known to be responsible for the regulation of innate immunity and inflammation. Both receptor-mediated forward signaling and ligand-mediated reverse signaling play important roles in these processes. In this review, we discuss the functional expression and roles of various reverse signaling molecules and pathways of TNFSF members in macrophages and microglia in the central nervous system (CNS). A thorough understanding of the roles of TNFSF ligands and receptors in the activation of macrophages and microglia may improve the treatment of inflammatory diseases in the brain and periphery. In particular, TNFSF reverse signaling in microglia can be exploited to gain further insights into the functions of the neuroimmune interface in physiological and pathological processes in the CNS.

## Introduction

Cell-to-cell communication, particularly for immune cells, occurs through either soluble mediators or direct contact. In cases of communication through soluble mediators, molecules, such as cytokines, chemokines, and hormones act in an autocrine, paracrine, or endocrine manner to stimulate cell surface receptors. In contrast, direct contact requires the interaction between cell surface molecules, such as cell adhesion molecules. Members of the tumor necrosis factor (TNF) superfamily (TNFSF) constitute a special class of molecules that are involved in both types of cell communication. TNFSF members are type II membrane proteins that are present on the cell surface or in intracellular compartments. In their membrane-bound form, TNFSF members can interact with their cognate TNF receptor superfamily (TNFRSF) members present on the cell surface or on adjacent cells. Cellular activation increases cell surface expression and secretion of homotrimeric forms of TNFSF members released from the cell surface. For TNF-α, this proteolytic cleavage is carried out by TNF-α-converting enzyme, a member of the ADAM family of metalloproteases. Released trimeric forms of these ligands then act as cytokines by interacting with their cognate receptors.

Increasing evidence has demonstrated that during direct contact within or among cells expressing TNFSF and TNFRSF members, signals are generated from the receptor part (forward signaling) as well as the ligand part (reverse signaling). Another interesting feature of the interactions between TNFSF/TNFRSF members is that there is substantial crosstalk among cognate ligand-receptor pairs, and some receptors can also be solubilized and released into the surrounding tissues, thereby serving as competitive inhibitors of ligand action on receptor-bearing cells (1, 2).

Macrophages are immune cells that express most members of the TNFSF and TNFRSF before and/or after activation. Macrophages perform immunoregulatory functions at sites of acute and chronic inflammation, pathogenesis, and tumorigenesis. Many of the functions of macrophages are mediated by TNFSF and TNFRSF members. Moreover, TNFSF and TNFRSF members are expressed in brain glial cells and mediate diverse biological effects, including neuroinflammation and cell death. Neuroinflammation is closely associated with diverse neuropathologies, such as CNS injury and neurodegenerative diseases (3). Recent evidence indicates that neuroinflammation is one of the major components of the disease mechanisms. Under pathological conditions, neuroinflammation and brain injury constitute a positive feedback loop that perpetuates damages in the nervous system. Brain glial cells, particularly microglia, play a pivotal role in these processes. Inflammatory and neurotoxic mediators produced from excessively activated microglia contribute to neurodegeneration. Intracellular and intercellular signaling of microglia has been proposed as a therapeutic target to dampen deleterious microglial activation and to protect neurons from microglial neurotoxicity (4–6). In that vein, TNFSF and TNFRSF members expressed in brain microglia may provide insights into the intercellular signaling of microglia, and shed light on the regulatory mechanisms of microglia-mediated neuroinflammation.

To date, many reviews have summarized the roles of forward signaling in various processes associated with normal immunity and the pathogenesis of cancer and other conditions. Therefore, in this review, we will focus on reverse signaling initiated from membrane-bound form of TNFSF with an emphasis on recent developments. Especially, members of TNFSF that are expressed in macrophage/microglial lineage cells, such as BAFF/APRIL, LIGHT, GITRL, FasL, TWEAK, and CD137L (4-1BBL), will be the main topic of this review.

## B-call Activation Factor of the TNF Family (BAFF)/a Proliferation-Inducing Ligand (APRIL)

**Figure 1:**
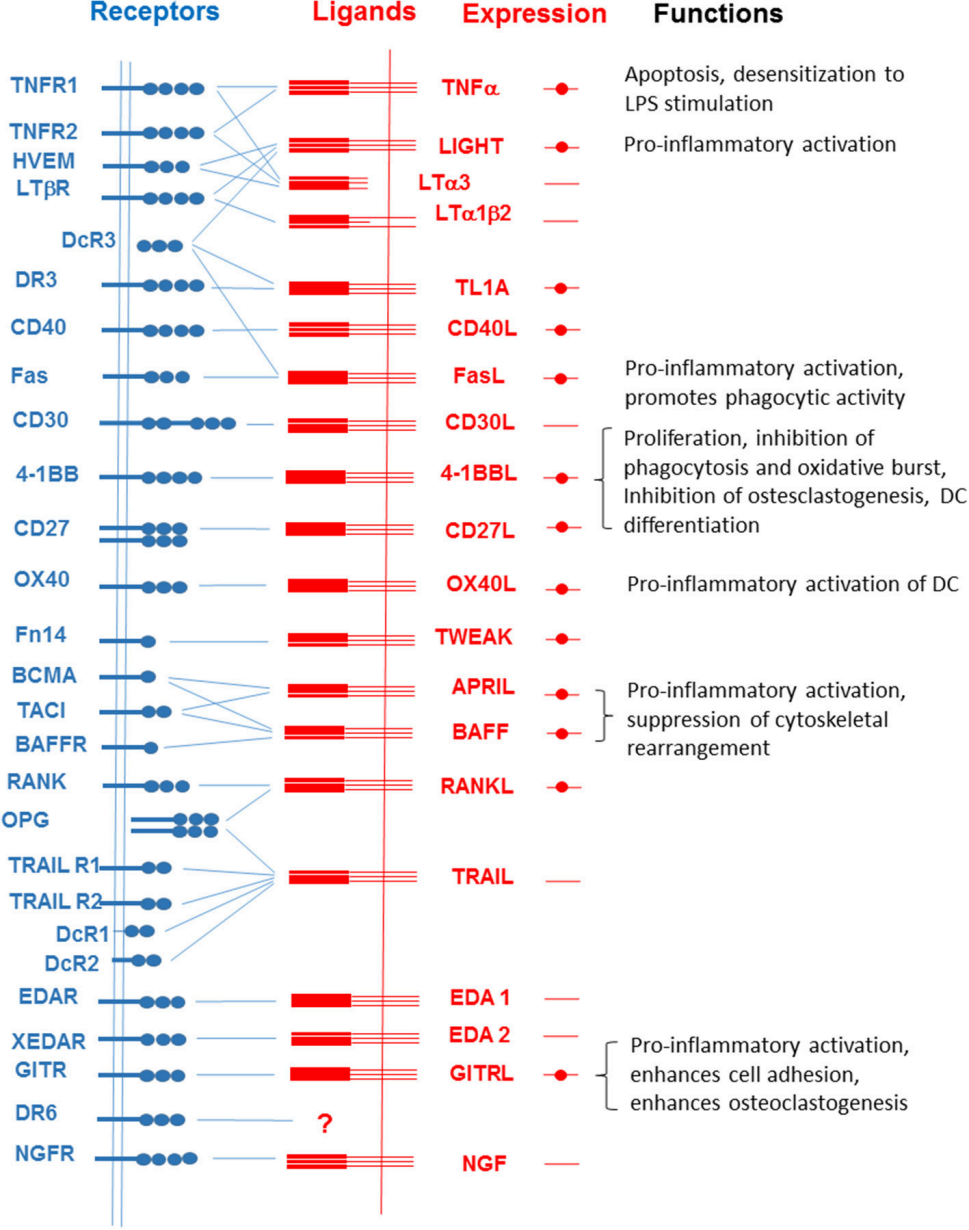
Interactions among members of the TNFSF and TNFRSF. Filled circles represent the expression of each member of the TNFSF in macrophages and microglial cells. Functions of reverse signaling in the activities of macrophages and microglial cells are listed on the right.

BAFF (also known as TALL-1, THANK, and TNFSF13B), a B-cell survival factor, and its close relative APRIL (also known as TNFSF13) are expressed in both membrane-bound and soluble forms in various cells lineages, including myeloid cells (monocytes, macrophages/microglia, neutrophils, and dendritic cells [DCs]), stromal cells within lymphoid organs, and osteoclasts (7–10). APRIL shares ~30% sequence identity with BAFF in the TNF domain (11). BAFF interacts with three types of receptors: transmembrane activator and a calcium-modulating cyclophilin ligand interactor (TACI), B-cell maturation antigen (BCMA), and BAFF receptor (BAFFR and BR3; Figure 1). These receptors can be found in lymphoid cells (i.e., B cells and plasma cells, but also in some subsets of T cells) and myeloid cells (8, 10). Although BAFF-R interacts with only BAFF, TACI, and BCMA interact with both BAFF and APRIL. Studies of APRIL and BAFF transgenic/knockout mice have revealed that these molecules are essential for B-cell survival, T-cell costimulation, autoimmune diseases, and cancer (8–11). Moreover, ligation of BAFFR activates B-cell survival through activation of the nuclear factor (NF)-κB pathway and downstream anti-apoptotic genes (12, 13). Although both BAFF and APRIL are required for B-cell maturation and survival, BAFF has major effects on pre-immune B cells, whereas APRIL acts on antigen-experienced B cells (14).

Both BAFF and APRIL contain a short cytoplasmic region of ~30 amino acids, a transmembrane domain (TMD), and a 200-residue extracellular domain consisting of a stalk and a TNF domain (11, 15–17). In macrophages, both are capable of inducing reverse signaling, which triggers inflammatory changes for the induction of various inflammatory mediators, including matrix-degrading enzymes and pro-inflammatory cytokines (18, 19). Treatment of either primary mouse macrophages or human macrophage-like cell lines with the TACI:Fc fusion protein or anti-BAFF/APRIL-specific monoclonal antibodies (mAbs) stimulates the cells to express various pro-inflammatory markers while suppressing cytoskeletal rearrangement associated with phagocytosis and transmigration (18–21). Furthermore, co-incubation with Ramos cells, which express both TACI and BCMA, results in pro-inflammatory activation of THP-1 cells in a BAFF- or APRIL-dependent manner, indicating that cell-to-cell interactions can stimulate BAFF- or APRIL-mediated reverse signaling (18, 21). These pro-inflammatory responses initiated by BAFF are mediated by the mitogen-activated protein kinase (MAPK) extracellular signal-regulated kinase (ERK) and NF-κB, and suppression of cytoskeletal rearrangement is mediated by phosphatidylinositol 3-kinase (PI3K)/AKT and Rac-1, a Rho-family GTPase. Interestingly, BAFF-mediated signaling shows significant crosstalk with Toll-like receptor (TLR) 4-mediated signaling such that simultaneous treatment with anti-BAFF mAbs and lipopolysaccharide (LPS) results in a synergistic response with respect to pro-inflammatory activation. The cellular response is mediated by PI3K/AKT and MAPK/Mitogen- and stress-activated protein kinase 1 (MSK1) pathways, which culminate at the formation of a trimeric complex containing NF-κB, cyclic AMP-response element binding protein (CREB), and CREB binding protein. This trimeric complex is responsible for the synergistic activation of NF-κB and, consequently, pro-inflammatory responses of the cell (22). The involvement of ERK in pro-inflammatory activation has been further confirmed in studies showing the existence of crosstalk between BAFF-mediated signaling and signals initiated from immune receptor expressed on myeloid cells 1 (IREM-1, CD300F) (23–25). IREM-1 is an immunoreceptor tyrosine-based inhibition motif (ITIM)-containing cell surface molecule that exerts its inhibitory effects through interaction of its ITIMs with SH2-containing tyrosine phosphatase (SHP)-1. Via its phosphatase activity, SHP-1 suppresses cellular signals associated with PI3K, Janus kinase 2, MAPKs, signal transducers and activators of transcription, and NF-κB (26, 27). Simultaneous stimulation of BAFF and IREM-1 results in suppression of BAFF-mediated ERK activation owing to IREM-1-mediated activation of SHP-1 (22). These findings indicate that it is necessity to re-evaluate the role of BAFF in diseases in which BAFF is overexpressed in macrophages.

**Figure 2:**
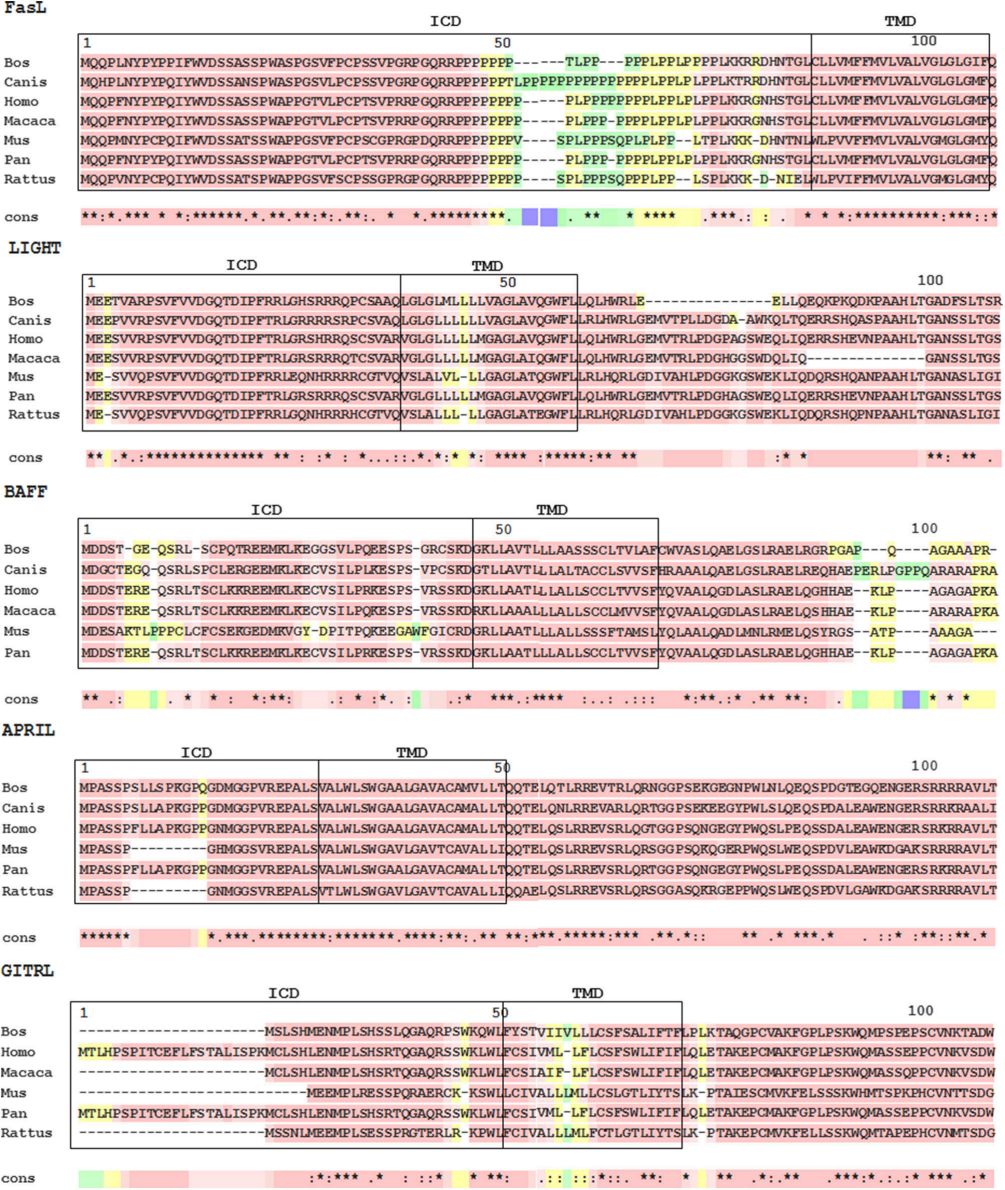
Multiple sequence alignments of TNFSF ligands. Protein sequences were aligned using NCBI homoloGene website (https://www.ncbi.nlm.nih.gov/homologene). Alignments of N-terminal sequences were constructed with T-coffee (The European Bioinformatics Institute [EMBL-EBI], https://www.ebi.ac.uk/). The transmembrane domain of APRIL was computationally predicted with Phobius (EMBL-EBI, https://www.ebi.ac.uk/). The pink area indicates amino acid conservation. ICD, intracellular domain; TMD, transmembrane domain.

Despite the similarities in their extracellular domains and receptors, BAFF and APRIL have quite different intracellular domains (ICDs; Figure 2). On the other hand, the ICD in each member is highly conserved in among different species, supporting the importance of their intracellular domains for the generation of reverse signaling.

BAFF/APRIL and their receptor systems are believed to be involved in the pathogenesis of various autoimmune diseases. Accordingly, serum BAFF levels have been shown to be abnormally upregulated in patients with systemic lupus erythematosus (SLE), rheumatoid arthritis (RA), and Sjögren's syndrome (28–31). The mAb-based therapeutic belimumab (LymphoStat-B) was approved by the US Food and Drug Administration for the treatment of SLE in 2011 (32). Belimumab was developed by screening of a phage-display library and consists of two heavy chains and two light chains with specificity against BAFF; this antibody blocks BAFF-mediated activation of its receptors and subsequent cellular activation (33). Although belimumab interacts with soluble BAFF and not with the membrane-bound form of BAFF (34), the antibody may crosslink with and thus activate the membrane bound-form of BAFF on cells of monocyte lineage. Another mAb-based therapeutic currently being evaluated in clinical trials is tabalumab (35), an anti-BAFF human mAb that has been reported to neutralize both membrane-bound and soluble forms of BAFF (36). Because tabalumab binds the membrane-bound form of BAFF, exploring whether this antibody induces reverse signaling from BAFF in various cell types could be beneficial for the future development of agents targeting BAFF or APRIL.

**Figure 3:**
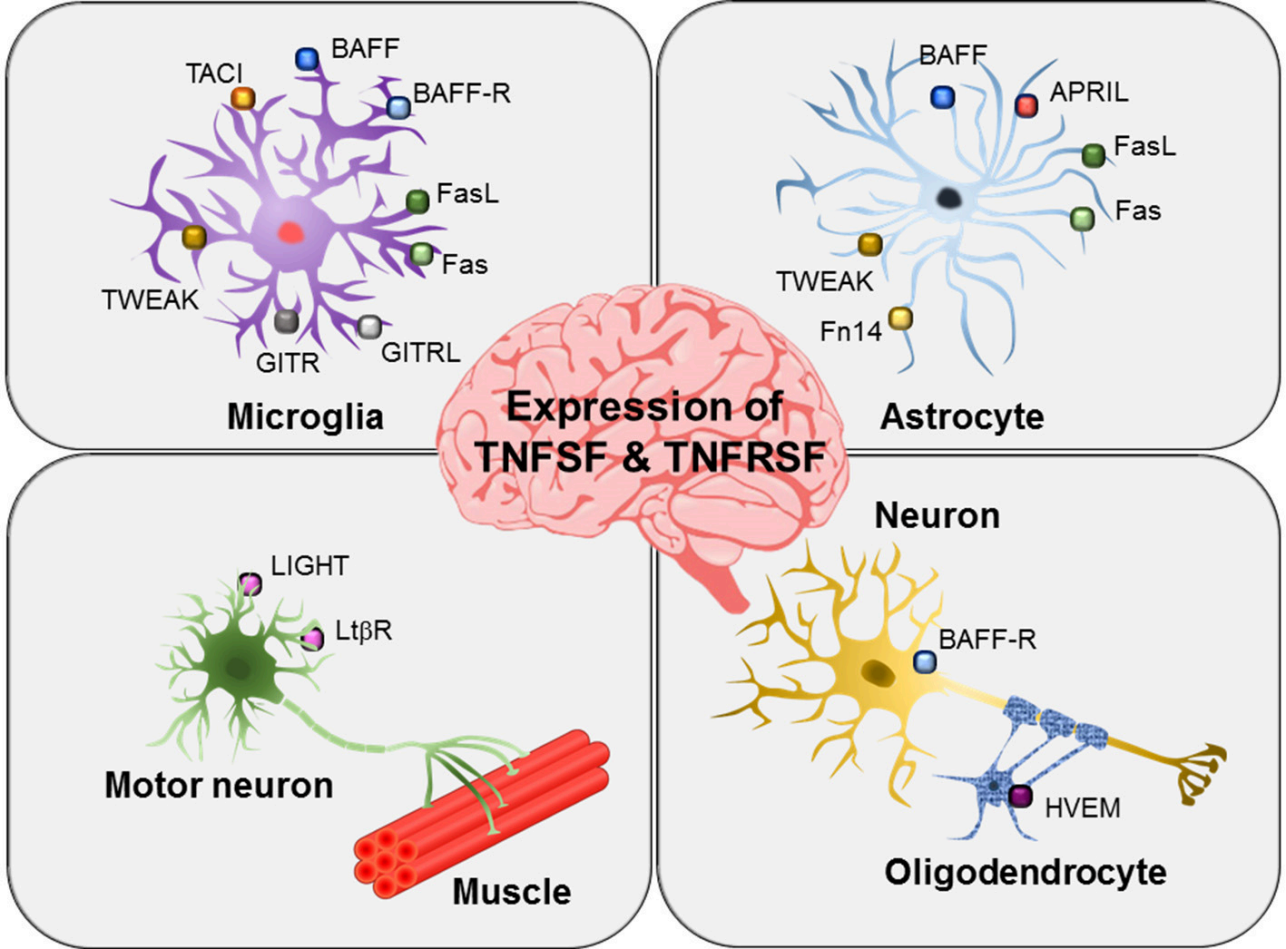
Expression of TNFSF and TNFSRSF members in brain glial cells and neurons. Different members of the TNFSF and TNFSRSF are expressed on microglia, astrocytes, oligodendrocytes, and neurons as indicated. In particular, motor neurons have been shown to express LIGHT and LTβR.

BAFF and its receptors are widely expressed in brain glial cells (Figure 3). Microglia express BAFF, BAFFR, and TACI (37). In contrast, astrocytes and neurons only express BAFF and BAFFR, respectively (38, 39). Microglial expression and release of BAFF is increased by ganglioside mixture treatment (37) and brain injury (38). In particular, cerebral ischemia and reperfusion injury enhance microglial BAFF and neuronal BAFFR expression, suggesting important roles of the BAFF/BAFFR interaction in brain injury conditions (38). Neuronal survival was promoted by BAFF/BAFFR ligation under ischemic stress conditions *in vitro* as well as middle cerebral artery occlusion *in vivo*. Interactions between microglial BAFF and neuronal BAFFR seem to exert neuroprotective effects in brain ischemia injury and may represent a promising therapeutic target for patients with stroke. BAFF released from microglia has been proposed to act on microglia themselves or B cells infiltrated into the brain to regulate central nervous system (CNS) inflammation (37). A previous study by Krumbholz et al. identified astrocytes as a main cellular source of BAFF in multiple sclerosis plaques, suggesting that BAFF produced by brain astrocytes may be involved in B-cell survival under inflammatory conditions (39). BAFF has also been reported to have a different functional role in experimental autoimmune encephalomyelitis (EAE); specifically, *BAFFR* gene-deficient mice show increased peripheral inflammatory cytokines and higher disease severity compared with wild-type animals, suggesting alteration of macrophage activation and immune responses in the absence of BAFFR (40).

Reverse signaling of BAFF has not been specifically investigated in microglia or other glial cell types. Nevertheless, the wide distribution of BAFF and its receptors in various neural cell types indicates that BAFF/BAFFR signaling may be important for interglial crosstalk or neuron/glia interactions.

APRIL has been shown to be expressed by astrocytes in areas of gliosis and by several glioblastoma cell lines (Figure 3) (41). Under inflammatory conditions, astrocytes act like microglia, producing pro-inflammatory cytokines, chemokines, and nitric oxide. Astrocytic expression of APRIL has been shown to be increased in the brains of patients with multiple sclerosis (41). Thus, APRIL expressed in reactive astrocytes may participate in the regulation of neuro-inflammatory responses and gliotic scar formation in multiple sclerosis and other pathological conditions. Notably, in this previous study, microglia were negative for APRIL expression. However, the role of APRIL in glioblastoma cells is still not clear.

Further evidence of the role of BAFF and APRIL in CNS inflammation was obtained from a marmoset monkey model of multiple sclerosis (42). Indeed, administration of antibodies against either human BAFF or APRIL delayed EAE development via different mechanisms.

## Light

The expression of LIGHT (also known as TNFSF14 or CD258) has been observed in activated T and B lymphocytes, monocyte/macrophages, granulocytes, natural killer (NK) cells, and DCs (43–46). LIGHT can interact with three types of receptors, i.e., herpes virus entry mediator (HVEM), lymphotoxin β receptor (LTβR), and decoy receptor (DcR3) (43, 47). HVEM or LTβR mediates LIGHT-induced T-cell costimulation and/or subsequent cytokine production (48–52), whereas DcR3, which is a soluble receptor without a TMD, works as a competitive inhibitor of LIGHT-induced cellular responses (43, 47, 53). HVEM (also known as TNFRSF14, LIGHTR, or TR2), which was initially identified as a cellular coreceptor for herpes simplex virus (HSV) entry (54), has a wide tissue distribution, including lymphoid tissues, and is expressed on peripheral blood leukocytes, such as T and B lymphocytes and monocytes (55, 56). Similar to other members of this receptor superfamily, HVEM stimulation leads to the activation of transcription factors, including NF-κB and activator protein (AP-1) (56). The expression of LTβR has been detected on endothelial, epithelial, and myeloid cells (57). LTβR functions as a mediator of cancer-associated inflammation (58, 59), regulator of lymphoid organ development (60, 61) and homeostatic stimulator of DC expansion (62, 63). LTβR-mediated signaling induces the classical NF-κB pathway via TNF receptor-associated factor 2/5 (TRAF2/5) (64, 65) or the non-canonical NF-κB pathway via TRAF3 (66, 67). LTβR can also interact with and be stimulated by LTα1β2, which is expressed on the surface of the cell. Because HVEM also interacts with the homotrimer of LTα (LTα3) (57, 64), there seems to be extensive crosstalk between LIGHT/HVEM and LT/LT receptor systems (Figure 1).

The possibility of LIGHT-mediated reverse signaling has been reported in T cells, in which stimulation of LIGHT has costimulatory effects; indeed, treatment with anti-LIGHT mAbs enhances responses induced by T-cell receptor ligation. These responses include cell proliferation, cytokine production, and cytotoxic activity via MAPK activation. Although treatment of mice with DcR3-Fc downregulates graft-vs.-host responses and ameliorates the rejection of mouse heart allografts, it is not clear whether these effects are mediated by direct stimulation of membrane-bound LIGHT or perturbation of LIGHT-induced activation events (68, 69). Reverse signaling in macrophage lineage cells was demonstrated when the human macrophage-like cell line THP-1 was treated with a LIGHT-specific agonistic mAb. Cells responded by inducing the expression of pro-inflammatory mediators, such as interleukin (IL)-8 and matrix metalloproteinase (MMP)-9 while suppressing phagocytic activity. The signaling pathway initiated by LIGHT is mediated by the MAPK ERK and by PI3K, leading to activation of the major inflammatory transcription factor NF-κB (70).

Sequence analysis of the LIGHT ICD indicated a high level of conservation among different species. However, there were no similarities with currently known protein motifs (Figure 2).

LIGHT/HVEM/LTβR expression has not been thoroughly investigated in brain microglia or astrocytes. Instead, oligodendrocytes have been shown to express HVEM (71), and motoneurons express LIGHT and LTβR (Figure 3) (72). In an amyotrophic lateral sclerosis animal model, interferon (IFN)-γ secreted from astrocytes was found to induce LIGHT/LTβR signaling in motoneurons, thereby stimulating non-cell autonomous neurotoxic pathways (72). Moreover, researchers found that astrocyte/neuron crosstalk contributed to the elimination of motoneurons expressing both LIGHT and LTβR under pathological conditions. Microglia may also participate in the non-cell autonomous motoneuron selective death pathways by communicating with astrocytes or motoneurons. The study further suggested that IFN-γ/LIGHT/LTβR pathways may be useful therapeutic targets in motoneuron disease.

Although less is known about the glial expression of LIGHT/HVEM/LTβR, a previous study by Mana et al. investigated the role of LIGHT in CNS inflammation (73). In the EAE model, LIGHT was found to be involved in restraining macrophages and microglia, thereby limiting disease progression and nerve damage. However, further studies are required to elucidate the cell type-specific roles of LIGHT in autoimmune CNS inflammation because only conventional LIGHT-deficient mice have been evaluated.

HVEM expression has been found in oligodendrocytes (71). This study, however, focused on the role of HVEM as a receptor for viral entry during HSV-1 infection in a human oligodendrocytes. They observed the colocalization of HVEM and nectin-1 with HSV-1 particles, implying that HVEM may be a major viral receptor functioning in these cells.

## Glucocorticoid-Induced TNFR-related Protein (GITR) Ligand (GITRL)

GITR (also known as AITR or TNFRSF18) was originally identified in activated T-lymphocytes, functioning as a regulator of T-cell receptor-mediated cell death (74). Later, its expression was detected in regulatory T cells (Tregs), effector T cells, macrophages, and microglia (75–79). The ligand of GITR (GITRL) is mainly expressed in immature and mature DCs, B cells, endothelial cells, macrophages, and microglia (75, 80, 81). Forward signaling initiated from GITR acts to costimulate CD25-effector T cells, which respond through proliferation and cytokine production. This GITRL-induced forward signaling is mediated TRAFs and NF-κB (82–85). In human macrophage-like THP-1 cells, ligation of GITR results in the expression of pro-inflammatory mediators via activation of MAPK and PI3K (77). The GITRL/GITR system has also been implicated in various processes, including suppression of CD4^+^CD25^+^ Tregs, antiviral and antitumoral responses, leukocyte extravasation, RA development, and chronic lung inflammation (79, 86–88).

Reverse signaling through GITRL has been the subject of intense investigations. GITR^−/−^ mice show decreased numbers of leukocytes in inflamed areas (89, 90), and treatment of experimental animals with GITR-Fc fusion protein ameliorates the symptoms of autoimmune or chronic inflammatory diseases (91). These effects may be due to induction of GITRL-mediated reverse signaling or blockage of GITR signaling (neutralizing GITRL). Additional studies have indicated that both of these mechanisms are possible. Some reports have favored the reverse signaling mechanism. For example, adherence of GITR^−/−^ murine splenocytes or HL60 human monocytic cells to endothelial cells was found to be enhanced when the cells were treated with the GITR-Fc fusion protein. Moreover, stimulation with GITRL triggers the upregulation of intracellular adhesion molecule (ICAM)-1 and vascular cell adhesion molecule-1 (80). In contrast, other reports have favored the neutralizing effect of the fusion protein. Indeed, analysis of a spinal cord injury model in GITR^−/−^ mice indicated that the GITR-Fc fusion protein failed to alter the disease severity in the knockout mice but decreased disease severity in wild-type mice (92).

Stimulation of murine primary macrophages or human macrophage-like THP-1 cells with GITR-Fc fusion protein or anti-GITRL mAbs induces pro-inflammatory mediators (e.g., MMP-9, IL-8, monocyte chemotactic protein-1, TNFα, and IL-β) and upregulates ICAM-1 expression. GITRL-mediated activation signals were found to be mediated by ERK and NF-κB (93). Treatment of murine monocytic cells with recombinant soluble GITR (rsGITR) in combination with IFN-γ results in synergistic induction of inducible nitric oxide (NO) synthase (iNOS), cyclooxygenase (COX)-2, and MMP-9. Analysis of the signaling mechanisms indicated the involvement of tyrosine phosphorylation and NF-κB (94–96).

Both GITR and GITRL are expressed at the same time in some cell types, particularly macrophages and microglial cells (75–77). Clusters of macrophages/microglia can be easily observed in lesion areas in atherosclerotic plaques, synovium of patients with RA, and amyloid plaques in Alzheimer's brain. Activation signals initiated from both the receptor and ligand, which can occur within one cell or among adjacent cells, may cause synergistic pro-inflammatory activation.

GITRL-mediated reverse signaling is also involved in osteoclastogenesis. When osteoclast precursors are treated with receptor activator of NF-κB ligand (RANKL) and macrophage colony-stimulating factor (M-CSF), the expression of both GITR and GITRL is induced. Additional treatment with rsGITR enhances osteoclastogenesis, which is blocked by neutralizing anti-GITRL antibodies. This effect is related to rsGITR-induced production of prostaglandin E_2_ via COX-2. Prostaglandin E_2_ then downregulates the steady-state level of osteoprotegerin (OPG), which has anti-osteoclastogenic effects (97).

**Figure 4:**
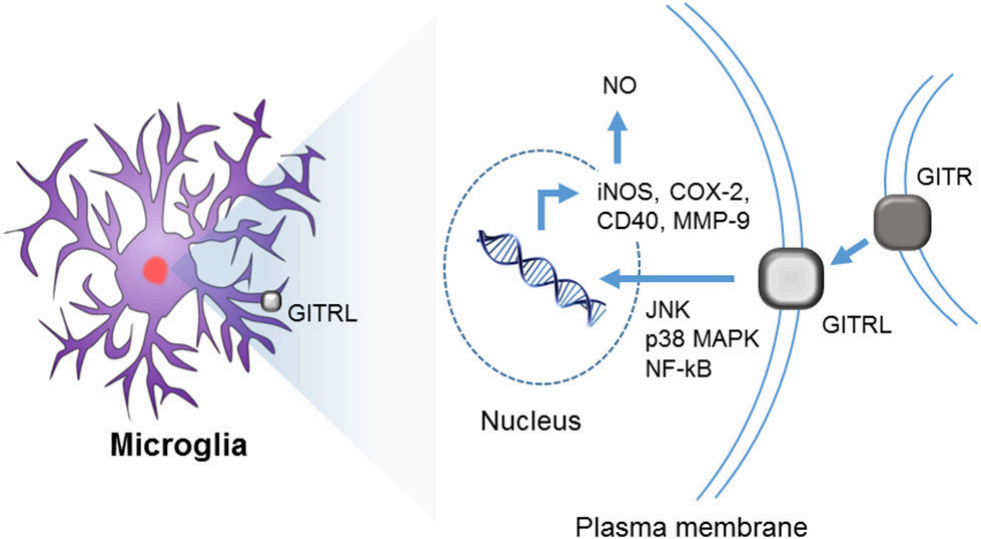
Reverse signaling through GITRL in brain microglia. Although both GITR and GITRL are expressed on microglia, only GITRL participates in the inflammatory activation of microglia. Upon ligation of GITRL, MAPKs (such as JNK and p38) and NF-κB are activated, and consequently, the expression of *iNOS, COX-2, CD40*, and *MMP-9* genes is induced with concurrent NO production.

Reverse signaling through GITRL has been well-characterized in microglia (Figures 3, 4). Hwang et al. first reported the expression of both GITR and GITRL in brain microglia and showed that reverse signaling through GITRL in microglia induces inflammatory activation, as determined by NO production and pro-inflammatory gene expression, such as iNOS, MMP-9, COX-2, and CD40 (Figure 4) (75). Furthermore, they demonstrated that GITRL-mediated microglial activation is executed by canonical inflammatory signaling, such as NF-κB and MAPK pathways. These results indicate that the GITR/GITRL system, particularly GITRL reverse signaling, may play a regulatory role in microglia-mediated neuroinflammation.

## Fas Ligand (FasL)

Fas (CD95), a type I transmembrane protein with characteristic cysteine-rich domains, works as a receptor for FasL (CD95L/CD178) (98, 99). Ligation of Fas induces caspase-dependent apoptotic cell death through its death domain, which is found in the ICD (100–103). Constitutive expression of Fas has been detected in many cell types, although FasL expression is restricted to CD4^+^ T helper cells, activated CD8^+^ cells, NK cells, and macrophages (104). Developmental stage-dependent expression of Fas during hematopoiesis has also been reported (105). Alternative splicing of *Fas* mRNA generates seven isoforms, which include soluble forms that can serve as DcRs (106). Activation of T cells leads to upregulation of cell surface expression of FasL, which then interacts with Fas on the same or adjacent cells. This interaction triggers apoptotic cell death, called activation-induced cell death (107). NK cells and CD8^+^ cytotoxic T lymphocytes use Fas/FasL interaction as one of the two main pathways that are responsible for their cytotoxic effector functions (108). Cells in immune-privileged sites and some tumor cells constitutively express FasL for the suppression of immune responses against them (109, 110).

In addition to its apoptosis-inducing properties during tissue injury and organ dysfunction (111–113), Fas also works as an enhancer of pro-inflammatory responses (114, 115) though caspase-independent and myeloid differentiation factor 88 (MyD88)-dependent signaling pathways (116–118). MyD88 serves as cross point for the crosstalk between the Fas-mediated signaling pathway and IL-1R1 and/or TLR4-mediated signaling pathways (119). Fas also enhances the proliferation of fibroblasts and T cells (120–122), which are involved in caspase activation without cell death (123–126). In THP-1 cells, treatment with anti-Fas mAbs or incubation with FasL-expressing cells results in pro-inflammatory activation of these cells through activation of ERK and NF-κB (127). These reports indicate that Fas-mediated signaling pathways are under complex regulation and provoke various responses in different cell types.

Unlike other members of the TNFSF, FasL has a long ICD containing around 80 amino acids with a high level of conservation across different mammalian species. The ICD of Fas contains two casein kinase I (CKI) binding sites and a proline-rich region that contains multiple binding sites for the SH3 domain (2). The CKI phosphorylation motif can be found in five other members of the TNFSF and is required for the FasL-mediated activation of nuclear factor of activated T cells (NFAT) and costimulation of T cells (104) (128). Possible interactions of this ICD with SH3-containing signaling adapters, such as Grb2, Fyn, and PI3K, have been reported (129, 130). The proline-rich sequence is also required for the storage of FasL in specialized secretory vesicles and the translocation of FasL to the plasma membrane upon activation (131). This intracellular localization appears to be regulated by the interaction of the proline-rich region with the SH3-containing adapter protein PSTPIP, which further interacts with the tyrosine phosphatase PTP-PEST (132).

Reverse signaling initiated from FasL, particularly via its proline-rich sequence, is involved in costimulation of CD8^+^ T cells, optimal thymocyte maturation, and antigen-driven proliferation of mature T cells (133–138). Upon activation, increasing fractions of FasL have been reported to be localized in lipid rafts, sphingolipid- and cholesterol-enriched dynamic membrane microdomains required for some of signaling and trafficking processes (133, 139). Along with increased localization in lipid rafts, activated FasL associates with SH3-containing proteins. The proline-rich domain is required for phosphorylation of FasL itself and other signaling molecules, including AKT, ERK, and c-Jun N-terminal kinase (JNK). These signaling events then activate transcription factors (NFAT and AP-1) and enhance IFN-γ production (1, 128, 133). Interestingly, FasL with an alteration in the proline-rich region (deletion of amino acids 45–54) abolishes its costimulation activity without affecting its death-inducing activity, indicating the separation of functional domains for different functions (128).

Although various functions of FasL have been well-documented in T cells, its roles in other cell types have not been extensively investigated. In breast cancer cell lines, IFN-γ treatment induces the translocation of pre-existing FasL to the cell membrane, and treatment with Fas-Fc fusion protein induces apoptotic cell death (140). In the human macrophage-like cell line THP-1, treatment with either anti-FasL mAbs or Fas-Fc fusion protein induces the production of pro-inflammatory mediators (e.g., MMP-9, TNF-α, and IL-8) and promotes phagocytic activity. This pro-inflammatory activation is mediated by MAPKs and NF-κB. In addition, FasL-mediated inflammatory activation is blocked by triggering of IREM-1 (141).

Fas and FasL are expressed in both microglia and astrocytes (Figure 3). As recently reviewed by Jha et al., microglia/astrocyte crosstalk constitutes an important component of neural cell communication, orchestrating a range of physiological and pathological processes in the CNS, such as brain development and neurological dysfunction (142). The bi-directional communication between microglia and astrocytes is mediated by either secreted or cell surface proteins. Fas/FasL expressed in microglia and astrocytes may mediate such crosstalk. Moreover, a previous study by Badie et al. suggested a role of the Fas/FasL interaction in microglia/glioma crosstalk (143). The authors reported that expression of the membrane-bound form of FasL is increased in the glioma environment and that FasL-expressing microglia may contribute to the local immunosuppressive environment of malignant glioma. However, the precise role of microglia in glioma biology remains unclear.

Fas is expressed at low levels and is upregulated upon TNF-α or IFN-γ treatment in primary mouse microglia cultures (144, 145). Moreover, Fas is expressed constitutively on astrocytes and is upregulated by treatment with IL-1, IL-6, or TNF-α. FasL is expressed on fetal and adult astrocytes and on microglia (144, 145). Thus, glial Fas/FasL may have a role in the induction of apoptosis in the CNS (144). Interestingly, however, the Fas/FasL interaction results in different signals in microglia vs. astrocytes. For example, Fas mediates cell death signaling in microglia, but transmits an inflammatory signal in astrocytes (145). Wang et al. have reported that astrocytic FasL mediates the elimination of autoimmune T cells in the CNS, contributing to recovery from EAE (146). This regulatory role of FasL expressed in astrocytes was demonstrated using glial fibrillary acidic protein/Cre FasL (fl/fl) mice in which the *FasL* gene was selectively deleted in astrocytes. In contrast, a study by Okuda et al. showed a tissue destructive role of FasL in the acute phase of EAE (147). When neutralizing antibodies against FasL were injected intrathecally, the disease severity was attenuated, and neuroinflammation and myelin damage were reduced in the CNS.

## TNF-like Weak Inducer of Apoptosis (TWEAK)

As a member of TNFRSF, fibroblast growth factor-inducible 14 (FN14) is expressed in various cell types, including lymphocytes, macrophages, endothelial cells, fibroblasts, and keratinocytes, particularly under conditions, such as inflammation and malignancy. Its ligand, TWEAK, is expressed in lymphocytes, macrophages, NK cells, renal tubular epithelial cells, and glomerular mesangial cells (148–150). Interestingly, both TWEAK and FN14 are widely distributed among many tissue types after exposure to inflammation (151). As a result, the TWEAK/FN14 system has been shown to be involved in inflammation, angiogenesis, cell proliferation, and apoptosis and in various diseases, including SLE, renal damage, RA, cancer, and conditions associated with cutaneous inflammation (152–154). FN14-mediated forward signaling leads to NF-κB activation (155, 156).

TWEAK-mediated reverse signaling has not been reported. However, a naturally occurring fusion protein between the ICD of TWEAK and the receptor binding domain of APRIL, called TWE-PRIL (157–159), has been reported. Analysis of APRIL^−/−^ mice (which lack APRIL and TWE-PRIL) demonstrated the involvement of TWE-PRIL reverse signaling in suppression of sympathetic axon growth and tissue innervation (160).

*TWEAK* mRNA expression has been detected in both microglia and astrocytes (161). FN14 expression has been reported in astrocytes; however, its expression has not been detected in brain microglia. The TWEAK/FN14 interaction has been implicated in CNS inflammation. Proliferation of FN14-expressing astrocytes is increased upon exposure to recombinant TWEAK protein. Moreover, *TWEAK* mRNA expression is enhanced in spinal cords during EAE, and disease severity is increased in transgenic mice overexpressing TWEAK. These results indicate that the TWEAK/Fn14 interaction in spinal glia is involved in CNS autoimmune inflammatory responses and can be targeted for EAE and MS therapy. Consistent with this, treatment of cultured human astrocytes with TWEAK increases ICAM expression and IL-6/IL-8 secretion, inducing reactive astrocyte-like characteristics (162). TWEAK also induces C-C motif chemokine ligand 2 (CCL2) release from astrocytes and endothelial cells in culture. Blockade of TWEAK/FN14 signaling inhibits TWEAK-induced CCL2 production and ameliorates EAE (163). Furthermore, administration of anti-TWEAK neutralizing antibodies reduces leukocyte infiltration and disease severity in EAE animals (164). These results are consistent with the pro-inflammatory and disease-promoting effects of TWEAK in CNS inflammation.

## CD137L (4-1BBL)

CD137 (4-1BB), originally identified as a T-cell costimulatory molecule, is expressed in activated T cells, B cells, NK cells, neutrophils, macrophages, and DCs and functions to promote their effector functions (165–167). The ligand of CD137 (CD137L, 4-1BBL) was found to be expressed in B cells, macrophages, and DCs (168, 169). Although CD137- or CD137L-knockout mice show no severe defects, they have a higher sensitivity to viral infection (170). Treatment with agonistic anti-CD137 antibodies or CD137L-Fc fusion protein results in expansion of tumor-specific T cells and ameliorates experimental autoimmune encephalomyelitis through modulation of the balance between Th17 and Tregs (171, 172).

For CD137L-mediated reverse signaling, most studies have been conducted using monocytes/macrophages. Stimulation of peripheral blood monocytes or bone marrow-derived macrophages with anti-CD137L mAbs or CD137-Fc fusion protein triggers a robust proliferative response, enhances cell adhesion, and/or stimulates pro-inflammatory activation associated with phosphotyrosine-mediated signaling (173, 174). This proliferation-inducing effect of CD137L has been reported to be mediated by the AKT/mammalian target of rapamycin (mTOR) pathway, resulting in reprogramming of glucose metabolism in a way that supports energy demand and biomass production. CD137L stimulation increases glucose uptake and upregulates enzymes involved in glucose transport/lysis and lactate production. Expression of genes involved in the pentose phosphate pathway and lipogenesis is also enhanced (175).

Bone marrow macrophages can be differentiated into osteoclasts by M-CSF and RANKL treatment. This osteoclastogenic process is inhibited by additional treatment with immobilized CD137L-Fc fusion protein or recombinant CD137 (176). Various experiments investigating this reverse signaling pathway have indicated that the signaling pathway is mediated by MAPKs, AKT, mTOR, PI3K, PKA, C/EBP, and CREB, resulting in induction of IL-6 and TNF expression (174, 177–179). Recombinant CD137 treatment also inhibits phagocytosis and oxidative burst (180). Interestingly, the extracellular domain of CD137L has been reported to directly interact with TNFR1, and this interaction appears to be required for CD137L-mediated reverse signaling. As a consequence, treatment of monocytes with TNF augments CD137-induced IL-8 expression, and inhibition of TNFR1 using TNFR1-neutralizing antibodies results in inhibition of CD137L-mediated responses, such as cell adhesion, apoptosis, CD14 expression, and IL-8 production (181). Using a two-hybrid system in a mouse macrophages, a novel transmembrane protein TMEM126A was found to interact with CD137L, and knockdown of TMEM126A was shown to abolish CD137L-mediated induction of tyrosine phosphorylation and pro-inflammatory cytokines (182). These results suggested the complex nature of CD137L-mediated reverse signaling. Further studies are needed to fully elucidate these mechanisms.

CD137L-mediated reverse signaling enhances DC maturation and potentiates the ability of DCs to stimulate T cells (180, 183). In contrast, a recent report showed that blockade of CD137L-mediated reverse signaling resulted in promotion of intratumoral differentiation of IL-12-producing CD103^+^ DCs and type 1 tumor-associated macrophages, which are required for the generation of IFN-γ-producing CD8^+^ T cells (184).

Most recently, CD137-CD137L signaling has been implicated in the hypothalamic interglial crosstalk under obese conditions (185). Mice fed with high-fat diet (HFD) showed an enhanced expression of CD137 and CD137L in the brain hypothalamus (186). Treatment of cultured glial cells with obesity-related molecules including free fatty acid and glucose promoted the expression of CD137 in astrocytes and CD137L in microglia, respectively (186). While forward signaling through CD137 in astrocytes increased their reactivity, reverse signaling through CD137L in microglia augmented the secretion of proinflammatory mediators, such as MCP-1. These recent findings suggest that CD137-CD137L signaling mediates microglia-astrocyte crosstalk in hypothalamic inflammatory responses under obese conditions, and CD137L reverse signaling in microglia might be a potential therapeutic target for the suppression of obesity-induced hypothalamic inflammation and related metabolic diseases.

## Reverse Signaling Initiated From Other TNFSF Members

The list of TNSF members that can induce reverse signaling is increasing as more studies focus on this aspect of the TNFSF/TNFRSF system. In the case of TNF, its ICD contains a nuclear localization signal sequence that can be liberated upon stimulation of the membrane-bound form of TNF (mTNF) with anti-TNF antibodies. This cleaved 10-kD fragment containing the ICD and TMD were found to be localized to internal membranes and nuclear fractions (187). Stimulation of mTNF with soluble TNFR increases intracellular calcium levels in RAW264.7 mouse monocytes (188). Additionally, soluble TNFR treatment causes changes in mTNF phosphorylation status, and casein kinase, which can phosphorylate the serine residues in the ICD of mTNF, has been implicated in this reverse signaling mechanism (188–190).

In monocyte/macrophage lineage cells, stimulation of mTNF with mAbs or soluble receptors leads to activation of MAPKs, particularly ERK, and the cells have been shown to be resistant to subsequent stimulation with LPS (191, 192). Other investigators have shown that stimulation of mTNF with anti-TNF antibodies results in internalization of the mTNF/anti-TNF complex into early endosomes and then lysosomes in macrophages and DCs (193). In addition, stimulation of synovial macrophages in RA joints with chimeric anti-TNF mAbs (infliximab) or soluble TNFR (etanercept) results in the induction of caspase-independent apoptotic cell death (194–196).

Some members of the TNFSF/TNFRSF show major crosstalk among ligand/receptor pairs. For example, DcR3, which is the counterpart of LIGHT, TNF-like ligand 1A (TL1A), and FasL, contains three conserved cysteine-rich domains characteristic of a TNFR (47, 197–199). Originally, DcR3 was thought to neutralize these members of the TNFSF through competition with their receptors. However, treatment of DCs with DcR3 modulates the differentiation and activation of DCs, which then directs naïve T cells to differentiate into a Th2 phenotype (200). In addition, monocytes and THP-1 cells respond to DcR3 treatment with induction of actin reorganization and enhancement of adhesion. Analysis of this reverse signaling revealed the involvement of protein kinase C (PKC), PI3K, focal adhesion kinase, and Src kinases (69). Additionally, the involvement of DcR3 in osteoclast development was also reported. When cells of monocyte/macrophage lineage were treated with DcR3, osteoclastogenesis was induced through MAPK signaling and TNF-α expression. These responses enhanced the development of osteoclast phenotypes, such as polynuclear giant morphology, bone resorption, and expression of tartrate-resistant acid phosphatase, CD51/61, and MMP-9 (201).

Of the three counterparts (LIGHT, TL1A, and FasL) of DcR3, reverse signaling has been reported for LIGHT and FasL, but not in TL1A. To date, there is no direct evidence supporting the generation of reverse signaling from TL1A. However, the expression of full-length TL1A has been shown to be correlated with the senescence of endothelial cells, and knockdown of TL1A expression has been shown to reverse the senescence phenotype (202). In a murine colitis model, cell surface expression levels of TL1A were found to be related to the suppressive activity of Tregs in a DR3-dependent manner, suggesting that the strength of signaling initiated from TL1A closely regulates Treg activity (203).

OX40 (CD134) is mainly expressed in activated T cells and acts as a costimulatory molecule for receiving activation and survival signals (204–206). The ligand of OX40 (OX40L) is mainly expressed in T and B cells, activated macrophages and DCs, and endothelial cells (207–209). The OX40/OX40L system has been implicated in T-cell costimulation, Treg generation, cell adhesion, and extravasation of T cells (210–214).

When B cells are co-incubated with OX40-expressing T cells or stimulated with soluble OX40, OX40L-mediated reverse signaling is induced, and the B cells undergo terminal differentiation into plasma cells. Because T cells are also activated through this interaction, the OX40/OX40L interaction appears to induce bidirectional signaling events (215, 216). A recent analysis of OX40L expression levels in B cells from patients with allergic rhinitis indicated that OX40L expression is positively correlated with allergic markers, such as serum levels of IgE and IL-4 (217).

In freshly isolated human blood DCs, mAb-mediated crosslinking of OX40L enhances CD40L-mediated expression of IL-12. In DCs derived from monocytes with IL-4 and granulocyte-macrophage CSF treatment, ligation of OX40L enhances the production of pro-inflammatory cytokines (e.g., TNF-α, IL-12 p40, IL-1β, and IL-6) and the expression of DC activation markers (e.g., CD83, CD80, CD86, CD54, and CD40) (218). Although the signaling pathway has not been elucidated, these data clearly support the role of OX40L-mediated reverse signaling in DC activation and maturation.

As a ligand of CD40, CD40L (CD154, gp39) is expressed in and activates T cells, B cells, DCs, macrophages, smooth muscle cells, endothelial cells, and platelets. CD40 expression has been detected in B cells, monocyte/macrophages, DCs, mast cells, fibroblasts, and endothelial cells (219–221). The CD40/CD40L system is important for activation of B cells and subsequent differentiation of these cells into plasma cells and stimulation of immunoglobulin class switching. In addition, this system is also involved in T-cell priming, T cell-mediated effector functions, macrophage/NK cell/endothelial cell activation, organ-specific autoimmune diseases, graft rejection, and atherosclerosis (222–225).

CD40L-mediated reverse signaling has been studied in CD40-knockout mice, in which defective germinal center development and antibody production were restored by soluble CD40 treatment. Additionally, reverse signaling was also found to be required for acquisition of B-cell activating potential (226). Although its ICD is only 22 amino acids, this region is highly conserved across various species and generates signaling through Lck, Rac1, MAPKs, and PKC in T cells (227–229). The presence of CD40 and CD40L in lipid rafts has also been reported and could explain the ability of these proteins to generate signaling (230, 231).

The expression of RANK has been detected mainly in osteoclasts and their precursors, DCs, and activated T and B cells. In addition, RANK expression has also been detected in a wide variety of tissues. The ligand of RANK (RANKL, also known as TRANCE) has been detected at osteoblasts, T cells, and stromal cells (232). The interactions between RANK and RANKL can be regulated by the decoy receptor OPG, which has affinity for both RANKL and TNF-related apoptosis-inducing ligand (233, 234). The RANK/RANKL/OPG system is involved in osteoclast differentiation/activation, bone remodeling, immune cell function, lymph node development, thermal regulation, and mammary gland development (235–238).

A few reports have provided evidence of RANKL-mediated reverse signaling. The expression of both RANK and RANKL has been detected in B chronic lymphocytic leukemia cells. Treatment of these cells with RANK-Fc fusion protein, but not with RANKL-Fc fusion protein, results in potent enhancement of IL-8 expression (239). Additionally, immobilization of RANK-Fc fusion protein augments IFN-γ secretion by Th1 cells in a p38 MAPK-dependent manner. Addition of RANK-Fc fusion protein during coculture of Th1 cells with antigen-presenting cells results in suppression of IFN-γ expression from Th1 cells, probably by blocking the interaction between RANK and RANKL (240). Moreover, osteoblasts, which express RANKL, regulate the differentiation and activation of osteoclasts and their precursors through the interactions of RANK and RANKL. Recent reports, however, have shown that reverse signaling initiated from RANKL is also possible. Soluble RANK treatment enhances p38-mediated mineralization of osteoblasts, which is abolished by knockdown of RANKL. When co-incubated with osteoclasts, osteoblasts respond by increasing p38 MAPK phosphorylation levels, and this response is blocked by abundant soluble RANKL (241).

CD30 is expressed in activated T and B cells and is a clinical marker for Hodgkin's lymphoma and related malignancies (242, 243). Interestingly, crosslinking of surface CD30 can activate latent human immunodeficiency virus in T cells (244, 245). CD30-mediated signaling has costimulatory effects in T and B cells, and serum levels of soluble CD30 serve as a prognostic marker of Hodgkin's disease and acquired immunodeficiency syndrome (246–248). The ligand of CD30 (CD30L, CD153) is expressed in activated T cells, B cells, and neutrophils. When peripheral blood neutrophils were stimulated with CD30L-specific mAbs or CD30-Fc fusion protein, cells responded by IL-8 production and oxidative burst. Peripheral blood T cells also responded to anti-CD3 and anti-CD30L antibody co-treatment by increasing metabolic activity, proliferation, and IL-6 production (249). According to a recent report, IgD^+^ IgM^+^ B cells express CD30L after activation with CD40L, IL-4, and specific antigen. Additional treatment with anti-CD30L antibodies or CD30-Fc fusion protein inhibits CD40-mediated signaling through TRAF2 and NF-κB, which results in reductions in class switch DNA recombination and subsequent production of IgG, IgA, and IgE (250).

The expression of CD27L (the ligand of CD27, CD70) has been detected in T cells, B cells, and NK cells. CD27 serves as a T-cell costimulatory molecule that enhances T-cell receptor-mediated signaling, proliferation, differentiation, and effector functions. The ligand of CD27 (CD27L, CD70) can be detected in lymphocytes, NK cells, and subsets of DCs (251–255). There have been numerous reports on the role of CD27-mediated forward signaling in the activation of T cells, B cells, and NK cells; however, few reports have demonstrated the existence of CD27L-mediated reverse signaling. In a study that explored the immune surveillance function of NK cells in cancer, B cells expressing cytoplasmic deletion mutant of CD27 were implanted in a B-cell acute lymphoblastic leukemia xenotransplant model. Expression of a truncation mutant in malignant cells increased the number of tumor-infiltrating IFN-γ-producing NK cells. Further analysis indicated that signaling mediated by CD70 on NK cells was transduced by AKT signaling and enhanced the survival and effector function of NK cells (256). In an earlier investigation, a subset of leukemic B cells was found to express CD27L, and stimulation with this ligand using specific mAbs resulted in enhanced cell proliferation. Furthermore, the proliferative response was synergistically enhanced by CD40 ligation (257).

## Future Perspectives

Although many studies have demonstrated the existence of reverse signaling initiated from TNFSF, it is still unclear how such signals are actually generated. The main reason for this is the short ICDs of these molecules, which usually lack any known signaling motifs. One exception is FasL, which contains several known protein motifs that can interact with multiple signaling adaptors. Although most members of TNFSF has short ICDs with lack of known signaling motifs, the high level conservation of ICD among various mammalian species and its uniqueness in each members of the TNFSF supports that these ICDs are involved in signal generation through yet unidentified mechanism. Bidirectional activation and possible crosstalk among signaling generated from these molecules are expected to generate a complex signaling network that regulates macrophage activity. Further studies are needed to explore this aspect of macrophage regulation.

Many antibody-based therapeutic approaches target the ligand part of TNFSF/TNFRSF system and aim for the blocking of receptor-mediated forward signaling. Some of these agents were proven to be effective for blocking the interaction between cognate ligand and receptor and thus the induction of forward signaling, which manifested in the alleviation of the severity of target disease(s). However, agents targeting the ligand itself or mimicking soluble receptor have the risk of activating membrane-bound form of ligands and, subsequently, generate the reverse signaling. These unwanted effects may degrade the therapeutic potential of the agents and may be able to explain some of the side effects observed during clinical trials. Additionally, it is also possible to develop agents that aim for the blockage of reverse signaling in the future.

Finally, the roles of the TNFSF/TNFRSF in CNS inflammation are complex and can be pro-inflammatory or anti-inflammatory depending on the context. Different members of the TNFSF and their receptors are expressed in distinct types of brain glial cells and neurons and exert context-dependent effects on neuroinflammation. Because the expression of these TNFSF members is dynamically regulated under a diverse CNS milieu, their functional roles may be modulated accordingly, with spatiotemporal regulation of the crosstalk of different TNFSF/TNFRSF members. Given the critical role of these TNFSF/TNFRSF members in regulating neuroinflammation, TNFSF/TNFRSF members and related signaling pathways can be potential drug targets for the control of neuroinflammation and the treatment of related diseases in the CNS. However, the benefits and challenges of such an approach must be weighed carefully given the multiple cell-cell interactions that might be affected. Compared with forward signaling of the TNFRSF, little is known about the reverse signaling through the TNFSF. Thus, further studies are needed to better understand reverse signaling pathways in brain glial cells and to determine the therapeutic applications of these pathways in the field of CNS inflammation. Finally, targeting forward and reverse signaling may have its own advantages and disadvantages depending on specific TNFSF and TNFRSF members; therefore, a combination of both is likely to be useful in the clinical settings.

## Author Contributions

All authors have made a substantial intellectual contribution to this work and approved submission of the manuscript. W-HL and KS formulated the focus of the review. DS and S-GL conducted the literature review and participated in the discussion. W-HL and KS wrote the manuscript.

### Conflict of Interest Statement

The authors declare that the research was conducted in the absence of any commercial or financial relationships that could be construed as a potential conflict of interest.
